# Influence of structure phase transition on the thermoelectric properties of Cu_2_Se_1−*x*_Te_*x*_ liquid-like compounds

**DOI:** 10.1039/d2ra04268a

**Published:** 2022-09-15

**Authors:** Trung Kien Mac, Thi Thu Ta, Huu Tuan Nguyen, Van Du Nguyen, Thi Lan Huong Pham, Van Thiet Duong, Tran Dang Thanh, Bach Thang Phan, Anh Tuan Duong

**Affiliations:** Faculty of Materials Science and Engineering, Phenikaa University Yen Nghia, Ha-Dong District Hanoi 12116 Vietnam tuan.duonganh@phenikaa-uni.edu.vn; Phenikaa Research and Technology Institute (PRATI), A&A Green Phoenix Group 167 Hoang Ngan Hanoi 10000 Vietnam; Faculty of Electrical and Electronic Engineering, Phenikaa University Hanoi 12116 Vietnam; Faculty of Biotechnology, Chemistry and Environmental Engineering, Phenikaa University Hanoi 10000 Vietnam; Graduate University of Science and Technology, Vietnam Academy of Science and Technology 18-Hoang Quoc Viet, Cau Giay Hanoi 10000 Vietnam; Institute of Materials Science, Vietnam Academy of Science and Technology 18-Hoang Quoc Viet, Cau Giay Hanoi 10000 Vietnam; Center for Innovative Materials and Architectures, Vietnam National University Ho Chi Minh City Vietnam

## Abstract

Copper chalcogenide Cu_2_(Se,Te) compounds are well known as typical p-type thermoelectric materials with a figure of merit (*ZT*) that can be optimized by the ratio of Se : Te. Here, by using the mechanical alloying and solid-state reaction methods, Te was substituted into Se sites within Cu_2_Se as the formula Cu_2_Se_1−*x*_Te_*x*_ (*x* = 0.1, 0.2, 0.25, and 0.3). The observed changes in structural phase, grain morphologies, and grain size were recorded by XRD and FE-SEM imaging with the appearance of the secondary phase of Cu_2_Te, with a Te content of *x* = 0.25. The layered structure morphology was observed more clearly at the high Te content. The electrical conductivity was greatly increased with enriched Te content while the maximum Seebeck coefficient was obtained in the Cu_2_Se_0.75_Te_0.25_ sample. Accordingly, a power factor value of up to 9.84 μW cm^−1^ K^−2^ at 773 K was achieved. The appearance of a Cu_2_Te phase with a Te content of 0.25 created a structural phase transition which results in a *ZT* value of 1.35 at 773 K in the Cu_2_Se_0.75_Te_0.25_ sample.

## Abstract 2

Copper chalcogenide Cu_2_(Se,Te) compounds are well known as typical p-type thermoelectric materials with a figure of merit (*ZT*) that can be optimized by the ratio of Se : Te.

## Graphical abstract

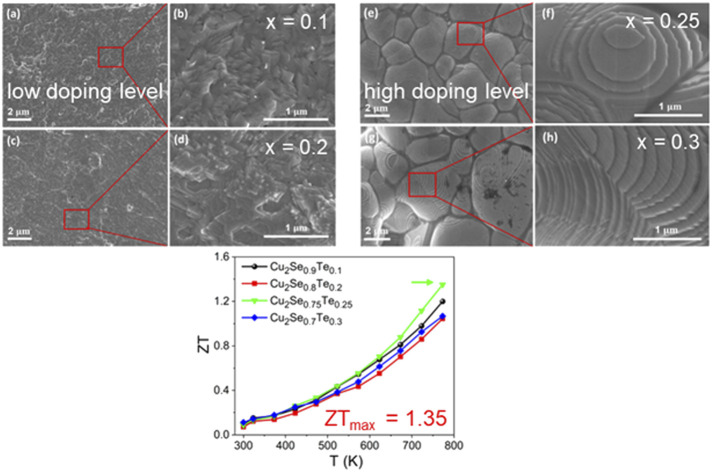

## 1. Introduction

Thermoelectric (TE) materials are of increasing interest because they can directly convert waste heat from furnaces, vehicle engines, factories, or human bodies to electric power without moving parts.^1^ The performance of TE materials is determined by the figure of merit (*ZT*), defined as *ZT* = *S*^2^*σT*/*κ*, where *S* is the Seebeck coefficient, *σ* is the electrical conductivity, *T* is the absolute temperature, and *κ* is the total thermal conductivity (*κ* = *κ*_E_ + *κ*_L_, where *κ*_E_ and *κ*_L_ are the electronic and lattice contributions, respectively).^2^ To maximize the *ZT* value of materials, larger power factor (*S*^2^*σ*) and lower thermal conductivity (*κ*) of materials are required.^3^ The Seebeck coefficient can be improved by some methods such as resonance doping,^4,5^ band convergence,^6^ hyperconvergence,^7,8^ Rashba splitting,^9^ band gap enlargement,^10,11^ and breaking of crystal mirror symmetry.^12^ The balance between low effective mass and high mobility of charge carriers can enhance their electrical conductivity.^3,13^ The thermal conductivity can be minimized by adding a number of interfaces and phonon scattering centers.^14^

Copper-base composites have been well-known as a high *ZT* value material. The copper chalcogenide compounds such as the Cu-base diamond-like compound (Cu_2_SnSe_3_, Cu_2_GeSe_3_, Cu_3_SbSe_3_, CuSbSe_2_, CuGaTe_2_ or CuInTe_2_,…) or Cu-base liquid-like compound Cu_2_(S, Se, Te) have high *ZT* value due to high electrical conductivity and low lattice thermal conductivity while other Cu-base such as the CuI compounds or CuI-based coordination polymer precursors exhibited high Seebeck coefficient.^15–19^ Among of the copper chalcogenide compounds, copper selenide (Cu_2_Se) has well-known as an excellent thermoelectric material due to its high *ZT* value, low cost, nontoxic, and tunable electrical transport properties. Belong to the group of liquid-like compounds, Cu_2_Se has a complex crystal structure with two main structural phases – α phase below 414 K and β phase formed above 414 K.^20^ In the high-temperature phase of Cu_2_Se, copper (Cu) ions are random located (liquid-like ions) inside the rigid sublattice of Se, leading to weak bonding and enhancing lattice phonon scatter.^16,21^ Until now, to increase the *ZT* value of Cu_2_Se, most researchers have focused on the control of doping elements and contents to modify electrical transport properties; nano-structuring to reduce thermal conductivity, and optimizing the growth method to control the structure and defect of materials for maximizing *ZT* value. Through the optimization of fabrication methods such as the hydrothermal, high-energy ball milling technique combined with spark plasma sintering (SPS), hot-pressing; and the control of the fabrication parameters (temperature and pressure), the *ZT* values of pure Cu_2_Se can achieve from 1.0 to 1.8 at around of 800–900 K.^22–27^ Fabrication of Cu_2_Se nanostructure leads to the formation of scattered interfaces, thus to reduce lattice thermal conductivity. On the other hand, under the nanoscale, quantum confinement effects enhance the electron density of states near the Fermi level, leading to the improvement of the Seebeck coefficient.^16^ The *ZT* values of Cu_2_Se nanostructure can be achieved above 2.0. For the doping method, the best TE performance with a *ZT* value of 2.62 was obtained at 756 °C for the bulk Cu_1.94_Al_0.02_Se.^28^ The good TE performance achieved by wet chemical method with the *ZT* from 0.9 to 1.6.^29,30^ Although the above manufacturing methods have resulted in well-improved TE properties of materials, they also have several limitations, which require expensive equipment and complex manufacturing protocol. Especially, for the melting process, which is necessary to rise to an extremely high temperature, takes more time, and the wet chemical method only produces a small amount of sample.

In the liquid-like compound of Cu_2_(S, Se, Te) group, the modification of S, Se, and Te elements content to maximize *ZT* value is attracting many research groups. Especially for control of Te contents in Cu_2_(Se, Te) material systems. In 2016, S. Butt *et al.* controlled Te doping contents (as Te-5%, Te-10%, Te-15%, and Te-20%, respectively in Cu_2_Se) and Te-10% doping in Cu_2_Se achieved the highest *ZT* value of 1.9 at 873 K.^31^ At lower 10% of Te doped Cu_2_Se, L. Yang *et al.* reported that the highest *ZT* value of 1.76 was observed at 850 K for Cu_2_Se_0.9_8Te_0.02_ sample while Y. B. Zhu *et al.* described the same composition of Cu_2_Se_0.9_8Te_0.02_ but using a different growth method, the *ZT* value could be achieved up to 1.25 at 773 K.^20,32^ K. Zhao *et al.* studied the thermoelectric properties of the series Cu_2_Se_1−*x*_Te_*x*_ (*x* = 0.2, 0.3, 0.5, and 0.7). The result showed that the crystal structure of the sample with Te content of 0.2 (Cu_2_Se_0.8_Te_0.2_) was nearly the same as Cu_2_Se, indicating no structural change.^33^ They also reported that the highest *ZT* value of 1.4 could be achieved for Cu_2_Se_0.7_Te_0.3_ at 1000 K with the Cu_2_Te phase observed. By using the chemical method, F.-H. Lin *et al.* reported the *ZT* value of 1.2 at 773 K and 1.4 at 920 K for the Cu_2_Se_0.96_Te_0.04_ sample.^34^ At around the transitional phase of the Cu_2_Se_1−*x*_Te_*x*_ (with Te contents between 0.2 to 0.3), the influence of Te content on the thermoelectric properties of these materials should be clarified.

Here, we focus on the Cu_2_Se_1−*x*_Te_*x*_ composition with Te contents of 0.1, 0.2, 0.25, and 0.3 by using a simple method, mechanical alloying combined with solid-state reaction (pressing and sintering). We found that, at 0.25 of Te content, the Cu_2_Te phase appeared, and a structural phase transition was observed. Especially, a new structural morphology was observed by FE-SEM images in the composition of Cu_2_Se_0.75_Te_0.25_ and Cu_2_Se_0.7_Te_0.3_. The highest *ZT* value of 1.35 was achieved at 773 K for the Cu_2_Se_0.75_Te_0.25_ sample.

## 2. Experimental

High purity powder of Cu (99.99%), Se (99.99%), and Te (99.99%) were weighed, according to the chemical compositions of Cu_2_Se_1−*x*_Te_*x*_ (*x* = 0.1, 0.2, 0.25, and 0.3). The stoichiometric elements were loaded into a tungsten carbide jar with tungsten carbide balls for mechanical alloying (MA) by a high-energy ball mill (FRITSCH Pulverisette 7, Germany) for 10 hours with the speed of 700 rpm. The nano-powder was loaded into a *Ø* 12 mm diameter mold and pressed under uniaxial pressure of 50 MPa at 523 K for 1 hour. The obtained samples were sintered at 873 K for 1 hour in an argon atmosphere. The crystal structure was investigated by X-ray diffraction (XRD) by using XRD EQUINOX 5000 Thermo Scientific, France with Cu-Kα radiation, *λ* = 1.54056 Å. Field-Emission Scanning Electron Microscopy (FE-SEM) was used to obtain the crystal morphologies. The electrical conductivity *σ* and Seebeck coefficient *S* were measured by a thermoelectric measurement system (Linseis-LSR3, Germany) using a four-probe contact method in a Helium atmosphere and a temperature range of 300 K to 773 K. The total thermal conductivity (*κ*) was calculated by the formula *κ* = *DC*_p_*d*. The thermal diffusivity (*D*) and the specific heat capacity (*C*_p_) of all bulk samples were measured by NETZSCH LFA 467 using the laser flash method in the Ar atmosphere. The sample densities (*d*) were obtained by the basic calculation *d* = *mV*^−1^, where *m* is the mass of the sample and *V* is its volume.

## 3. Results and discussion

**Fig. 1:**
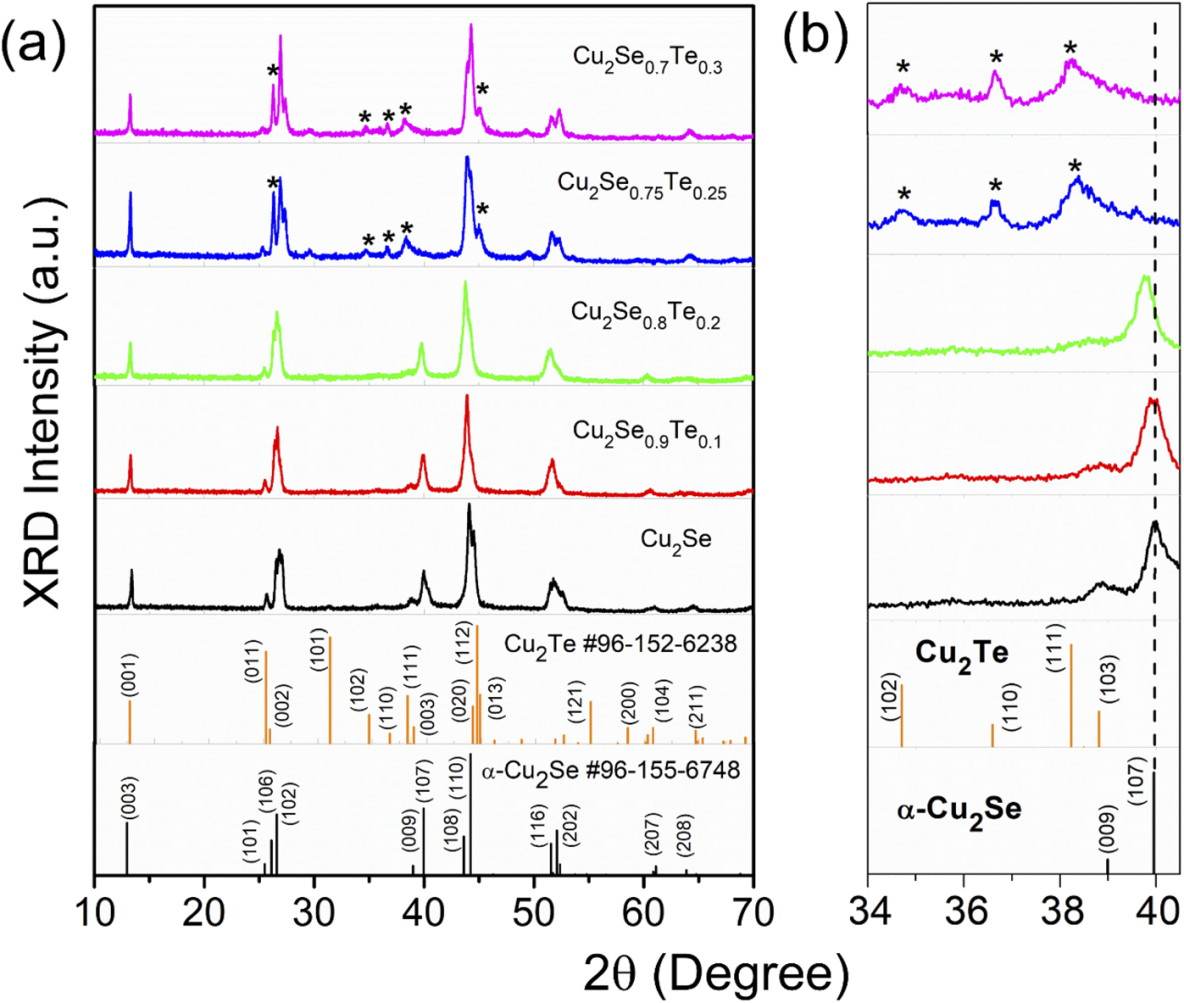
The room temperature X-ray diffraction of (a) Cu_2_Se_1−*x*_Te_*x*_ (*x* = 0; 0.1; 0.2; 0.25; and 0.3) and standard cards of α-Cu_2_Se and Cu_2_Te; (b) larger view of 2*θ* range from 34 to 40.5°.

The crystal structure of bulk Cu_2_Se_1−*x*_Te_*x*_ (*x* = 0.1, 0.2, 0.25, and 0.3) was observed by XRD patterns as shown in Fig. 1, corresponding to with two standard identification patterns of α-Cu_2_Se (card #96-155-6748) and Cu_2_Te (card #96-152-6238). At low Te content samples (Cu_2_Se_0.9_Te_0.1_ and Cu_2_Se_0.8_Te_0.2_), the XRD patterns were well-matched with trigonal α-Cu_2_Se (space group *R*3̄*m*). However, the XRD peaks shifted to a lower 2*θ* value with an increase of Te content, indicating that Te atoms substituted to Se sites in the Cu_2_Se lattice. A structural transformation of Cu_2_Se_1−*x*_Te_*x*_ was clearly observed at the proposed samples with the higher Te contents of *x* = 0.25 and 0.3. Particularly, the α-Cu_2_Se (107) diffraction peak at 2*θ* around 40° disappeared, meanwhile, a secondary phase of Cu_2_Te appeared (the Cu_2_Te peaks were marked (*) in XRD patterns), suggesting the formation of Cu_2_Se/Cu_2_Te hybrid structure at a Te content of 0.25. The structural phase transition of Cu_2_Se_1−*x*_Te_*x*_ was also observed by K. Zhao *et al.* with *x* ≥ 0.3.^33^ The enlarged patterns in the 2*θ* range of 34–40.5° were highlighted in Fig. 1b. The left shift of XRD peaks with increasing Te content indicated an expansion of lattice parameters.

**Fig. 2:**
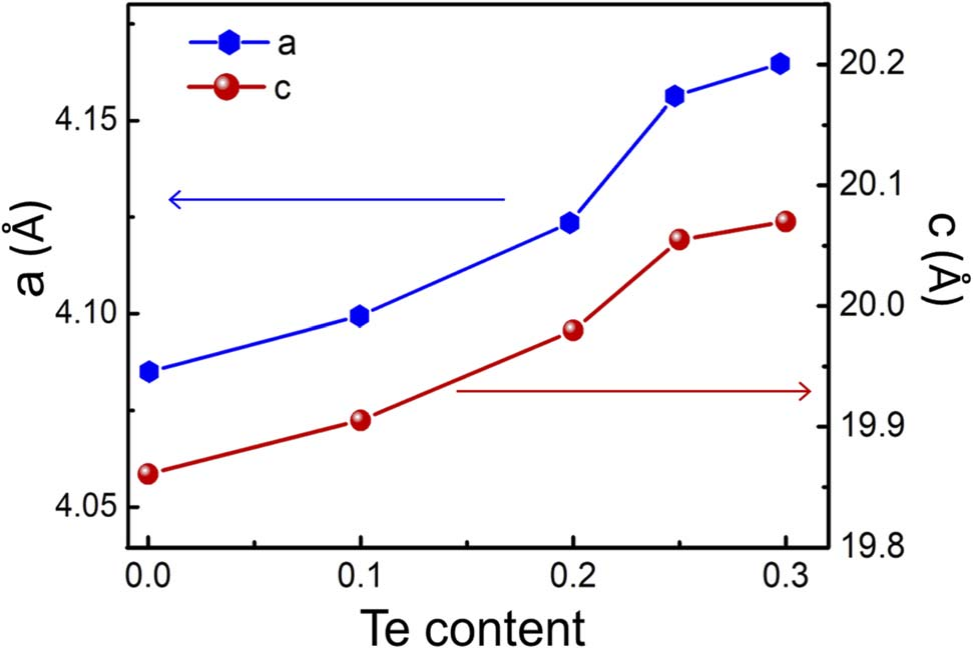
The increase of “*a*” and “*c*” lattice parameters with the increasing Te content.

Fig. 2 demonstrates the increasing of *a* and *c* lattice parameters from 4.08 Å to 4.16 Å and 19.86 Å to 20.07 Å, respectively with Te content from *x* = 0 to *x* = 0.3. It can be explained by the substitution of the larger ionic radius of Te (0.221 nm) than that of Se (0.198 nm). The tellurium concentration gradually increased, and Te elements replaced the Se position until the saturation state was reached (*x* = 0.25). The leftover Te formed in the Cu_2_Te phase, which leaded to a change in the XRD patterns and the morphological structure (discuss later) of the proposed samples.

**Fig. 3:**
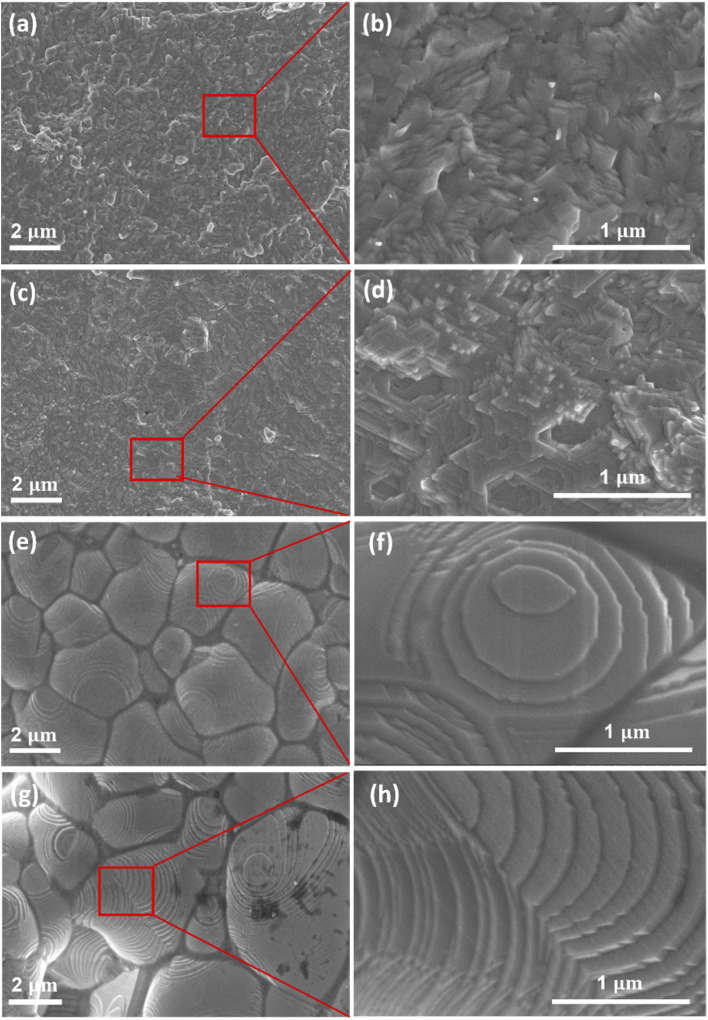
FE-SEM images show the morphologies of Cu_2_Se_1−*x*_Te_*x*_ (a) *x* = 0.1; (c) *x* = 0.2; (e) *x* = 0.25; (g) *x* = 0.3; (b), (d), (f) and (h) are higher magnification of the red square in (a), (c), (e) and (g), respectively.

Surface morphology of Cu_2_Se_1−*x*_Te_*x*_ (*x* = 0.1, 0.2, 0.25, and 0.3) was observed by Field-emission scanning electron microscopy (FE-SEM) as shown in Fig. 3. The typical layer structure of samples was remarkably formed with the increasing Te doping content. Especially in samples with higher Te content (*x* = 0.25 and 0.3), the grain morphologies and grain boundaries markedly changed. The average grain size of Cu_2_Se_0.75_Te_0.25_ and Cu_2_Se_0.7_Te_0.3_ was around 2.5 μm and 3.4 μm, respectively. A lamellar microstructure with an average thickness of a few nm was clearly observed in the high content of Te samples. The change of morphology and structure induces the improvement of electrical conductivity, which was discussed below.

**Fig. 4:**
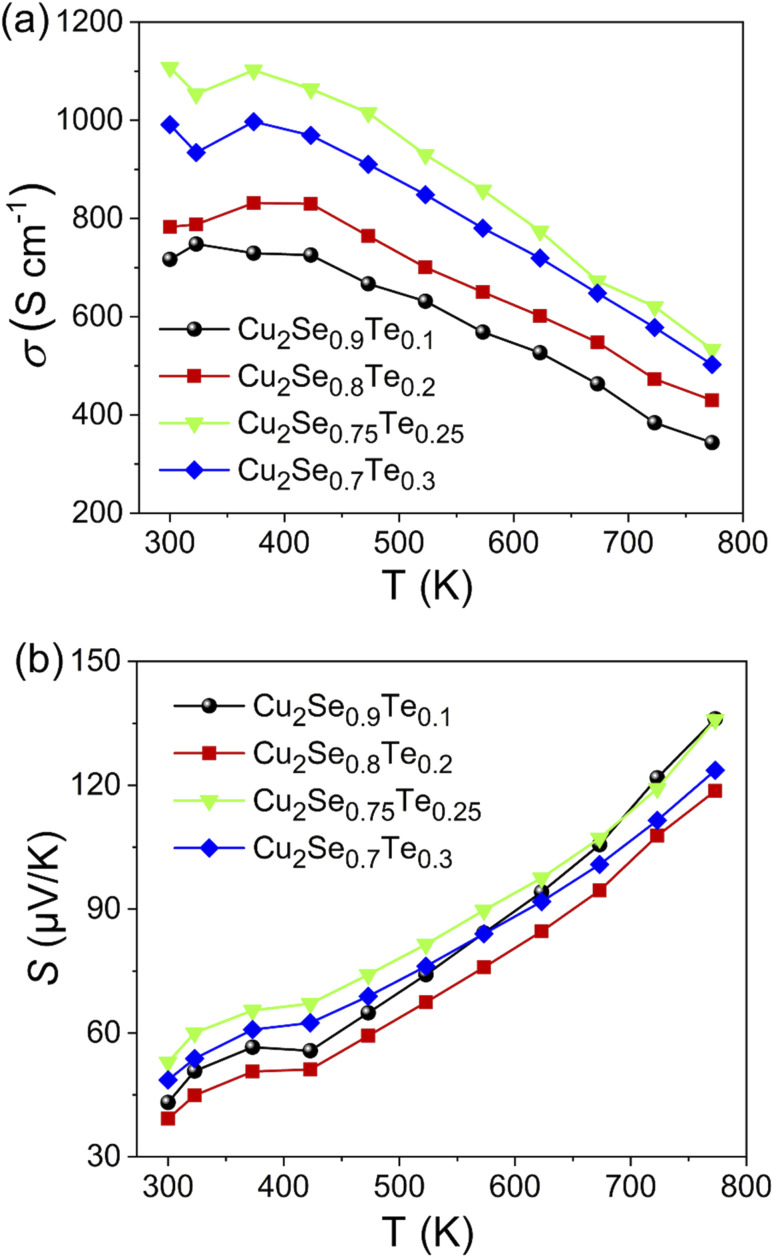
Temperature-dependent electronic transport properties of Cu_2_Se_1−*x*_Te_*x*_ (*x* = 0.1; 0.2; 0.25; 0.3). (a) Electrical conductivity (*σ*), (b) Seebeck coefficient (*S*).

The temperature dependence of electrical conductivity (*σ*) of Cu_2_Se_1−*x*_Te_*x*_ (*x* = 0.1, 0.2, 0.25, and 0.3) was shown in Fig. 4a. The electrical conductivity of all samples decreased with higher temperature, indicating that all samples showed a degenerate semiconductor. An anomalous point at around ∼400 K of electrical conductivity data is attributed due to the phase transition from α-phase to β-phase of Cu_2_Se.^35,36^ The *σ* value of all samples as a function of temperature above 400 K was decreased, indicating a typical degenerate semiconducting behavior. Thus, with an increasing measuring temperature, there are fewer charge carriers generated while the contribution of the decrease in carrier mobility is large, leading to a decrease in electrical conductivity. Besides, the electrical conductivity greatly increases with the increasing Te content. It can be explained by the higher intrinsic electrical conductivity of Cu_2_Te is greatly higher than that of Cu_2_Se.^16,20,33,37,38^ The highest value of electrical conductivity was observed in the Cu_2_Se_0.75_Te_0.25_. It is around 54% higher than that of Cu_2_Se_0.09_Te_0.0.1_ in the temperature range from 300 K to 773 K. The light-decreasing electrical conductivity of the Cu_2_Se_0.7_Te_0.3_ sample could be explained by the reduction of Cu^2+^ ions due to the Cu_2_Te phase formation. K. Zhao *et al.* also observed a decreasing in the carrier concentration of Cu_2_Se_0.7_Te_0.3_, compared to other doping samples.^33^

Seebeck coefficient as a function of temperature for all samples was presented in Fig. 4b. The positive Seebeck coefficient sign of all samples indicates that dominant charge carriers were holes and all samples showed p-type semiconducting behavior. As a type of degenerate semiconductor, the Seebeck coefficient of samples increased with an increase in temperature due to the electron–phonon scattering. In the temperature range from 300 K to 773 K, the maximum Seebeck coefficient of 135 μV K^−1^ was achieved in Cu_2_Se_0.75_Te_0.25_ sample, which can be related to the local thermal gradient due to the difference in the thermal conductivity of Cu_2_Te and Cu_2_Se.

**Fig. 5:**
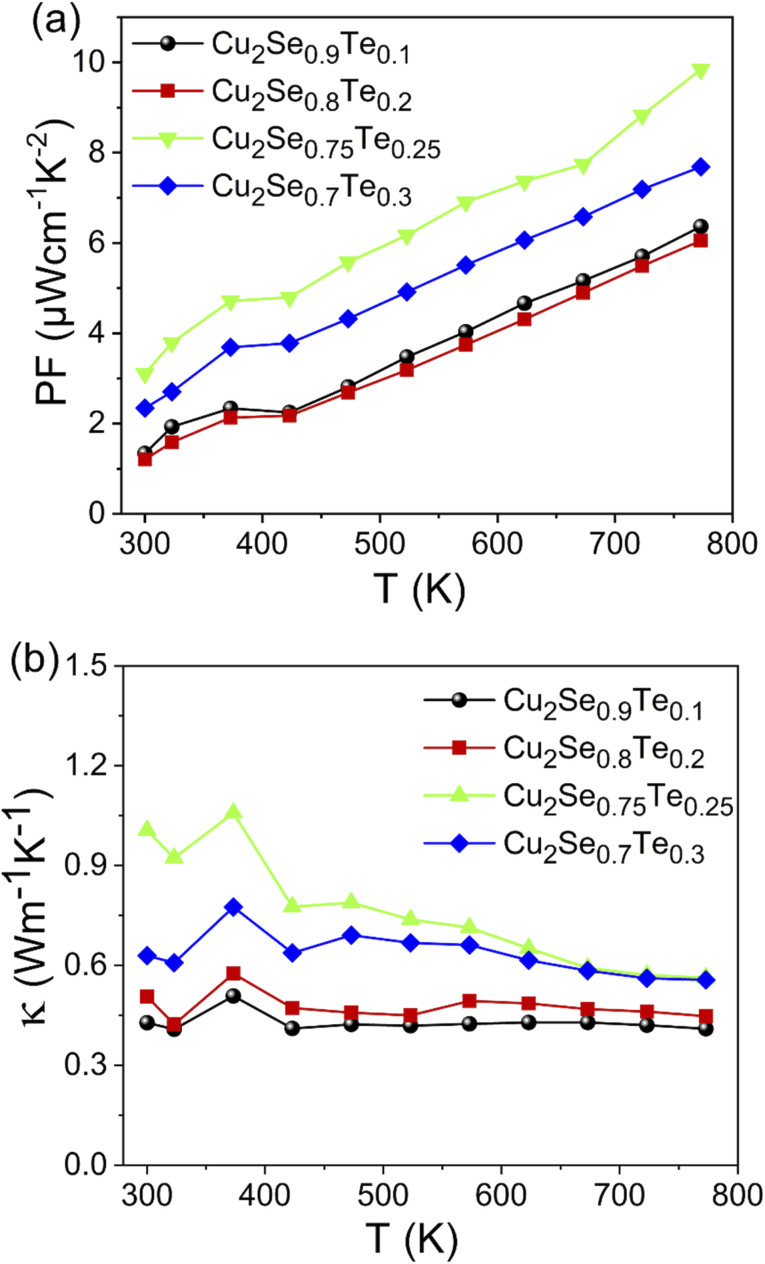
The temperature dependent (a) power factor and (b) thermal conductivity of Cu_2_Se_1−*x*_Te_*x*_ (*x* = 0.1; 0.2; 0.25; 0.3).

The thermoelectric power factor was calculated by *S*^2^*σ* as shown in Fig. 5a. The electrical conductivity increased with increasing Te content while the Seebeck coefficient was still high, resulting in the highest power factor of 9.84 μW cm^−1^ K^−2^ at 773 K in Cu_2_Se_0.75_Te_0.25_ sample. The non-linear PF at around 400 K was due to the phase transition from α-phase to β-phase of Cu_2_Se.

The temperature-dependent total thermal conductivity (*κ*) of Cu_2_Se_1−*x*_Te_*x*_ samples was presented in Fig. 5b. Those Te-doped Cu_2_Se samples also possessed very low total thermal conductivity, according to what we initially envisioned based on their natural liquid-like structures. As a general thermoelectric semiconductor, *κ*_L_ decreases due to the enhanced phonon scattering. Furthermore, combined with the trend of the electrical conductivity, it could be concluded that the carrier thermal conductivity did not have much change. Thus, as the measuring temperature increased, the total thermal conductivity decreased, except for the anomalous point near 400 K, where the phase transition (Cu_2_Se) occurred. Additionally, above 400 K, the thermal conductivity of Cu_2_Se_0.75_Te_0.25_ and Cu_2_Se_0.7_Te_0.3_ samples decreased while it was almost maintained in the samples with low Te (*x* = 0.1 and 0.2) with increase in measuring temperature. This phenomenon could be explained *via* the decrease of the lattice thermal conductivity at the synthetic samples with high amount of Te-doped. In contrast, the slight decrease in thermal conductivity at the lower Te-doped samples was due to the balance between *κ*_E_ and *κ*_L_. Further, the increase in thermal conductivity with increasing Te content also indicates that the phase transition arising from the formation of the Cu_2_Te phase contributed to the increasing thermal conductivity. It needs to be stressed that the thermal conductivity of Cu_2_Te was higher than that of Cu_2_Se. So, the formation of Cu_2_Te in samples impacted to the increasing thermal conductivity.

**Fig. 6:**
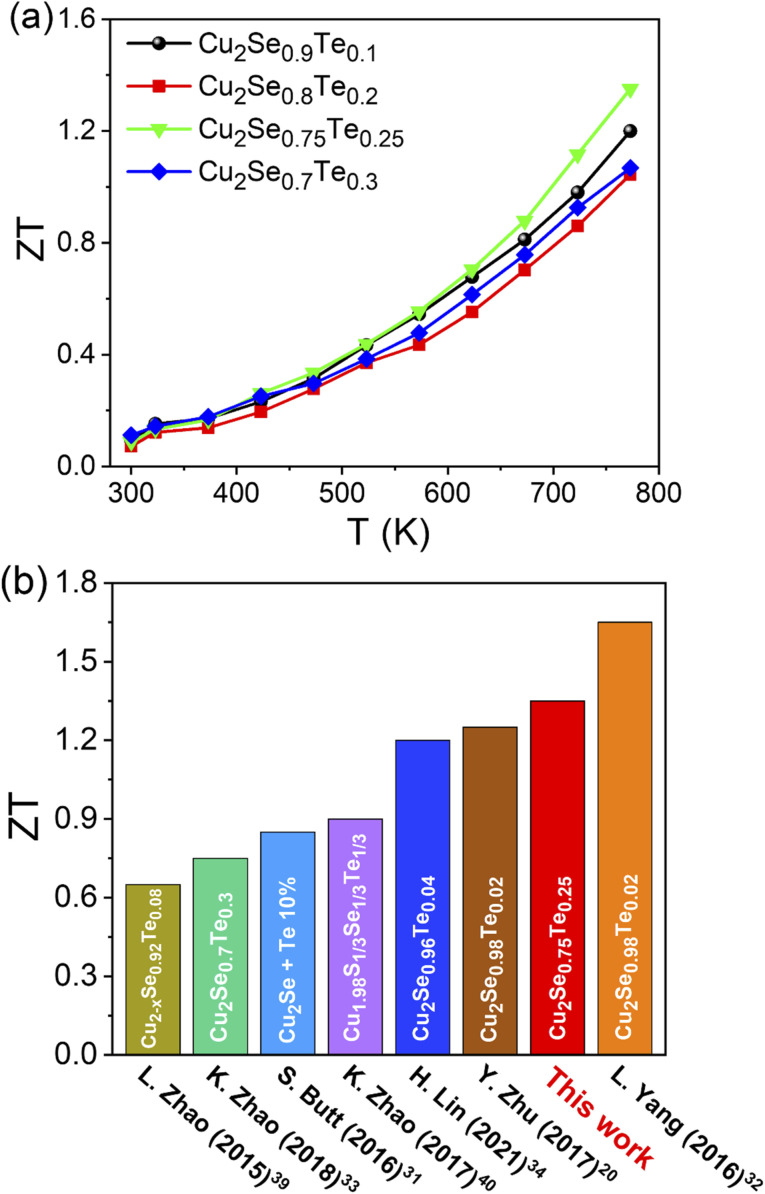
(a) The thermoelectric figure of merit (*ZT*) of Cu_2_Se_1−*x*_Te_*x*_ (*x* = 0.1; 0.2; 0.25; 0.3) and (b) comparison of *ZT* (*T* = 773 K) for present work with that reported in typical literatures of Te-doped copper selenide materials.

The temperature dependence thermoelectric figure of merit (*ZT*) of Cu_2_Se_1−*x*_Te_*x*_ (*x* = 0.1, 0.2, 0.25, and 0.3) was shown in Fig. 6a. The *ZT* values of all samples were increased with increasing temperature. With the balance of *σ*, *S*, and *κ* values, the highest *ZT* of ∼1.35 was obtained at Cu_2_Se_0.75_Te_0.25_, which owned the great power factor (9.84 μW cm^−1^ K^−2^ at 773 K) and ultra-low thermal conductivity (0.56 W m^−1^ K^−2^ at 773 K). At below 800 K, the *ZT* value in this work is almost higher than those of the pristine p-type Te-doped copper selenide materials in some previous reports under the different Te doping contents and synthetic method (Fig. 6b).^20,31–34,39,40^ In the Te contents range from *x* = 0.2 to 0.3, we found that the transition phase point at Te of 0.25 achieved the highest *ZT* value, compared with other Te contents. K. Zhao *et al.* investigated the thermoelectric properties of Cu_2_Se_1−*x*_Te_*x*_ (*x* = 0 to 1) solid solutions and they found the highest *ZT* value with *x* = 0.3 but the series of samples of *x* = 0.25 was missed.^33^

## 4. Conclusions

In summary, the Cu_2_Se_1−*x*_Te_*x*_ (*x* = 0.1, 0.2, 0.25, and 0.3) compounds have been successfully prepared by mechanical alloying combined with a solid-state reaction method. The structural phase transition of Cu_2_Se_1−*x*_Te_*x*_ was observed at Te content of 0.25 which obtained the highest *ZT* value of 1.35. A phase transition at 400 K from the α → β of all samples has been observed and confirmed using the measured results of electrical and thermal properties. *Via* the XRD and FE-SEM results, the change in crystal structure and morphology was observed according to Te content. By a more simple synthetic method, the obtained *ZT* value in this study has higher than that of almost previous study results. More interestingly, the optimization of fabrication parameters of Cu_2_Se_0.75_Te_0.25_ can lead to enhancing the *ZT* value of this material.

## Author contributions

T. K. Mac, and T. T. Ta, synthesized the Cu_2_Se_1−*x*_Te_*x*_ samples and performed the electrical conductivity and Seebeck coefficient experiments. V. D. Nguyen measured thermal conductivity, H. T. Nguyen, V. T. Duong, and T. L. H. Pham edited the manuscript and performed FE-SEM experiments. T. D. Thanh performed XRD experiments and edited the manuscript. B. T. Phan edited the manuscript. T. K. Mac wrote the paper with discussion and comments from all the authors. A. T. Duong supervised the project.

## Conflicts of interest

There are no conflicts to declare.

## Supplementary Material
